# *Mycoplasma pneumoniae*-Induced Rash and Mucositis (MIRM) Mimicking Behçet's Disease and Paraneoplastic Pemphigus (PNP)

**DOI:** 10.1155/2022/1013922

**Published:** 2022-08-22

**Authors:** Hanish Jain, Garima Singh, Timothy Endy

**Affiliations:** ^1^Division of Infectious Diseases, Department of Medicine, SUNY Upstate Medical University, Syracuse, NY, USA; ^2^Department of Medicine, Saint Vincent Hospital, Worcester, MA, USA; ^3^SUNY Upstate Medical University, Syracuse, NY, USA

## Abstract

MIRM is an uncommon entity characterized by prominent mucositis, usually with sparse cutaneous involvement. The diagnosis of MIRM can be challenging due to the lack of awareness amongst clinicians. Patients with Behçet's disease usually present with recurrent and painful mucocutaneous ulcers, while other clinical manifestations of the disease are more variable. Here, we describe an interesting case of MIRM mimicking Behçet's disease and PNP highlighting the overlapping manifestations and diagnostic challenges.

## 1. Introduction

MIRM was coined recently in 2015 to distinguish the mucocutaneous disease associated with mycoplasma from the Stevens-Johnson syndrome/toxic epidermal necrolysis (SJS/TEN) spectrum and erythema multiforme (EM) [1]. Behçet's syndrome is a rare disease characterized by recurrent oral aphthae and any of several systemic manifestations including genital aphthae, ocular disease, skin lesions, gastrointestinal disease, neurologic disease, vascular disease, and arthritis. Most clinical manifestations of Behçet's syndrome are believed to be due to vasculitis [2]. PNP is an often fatal autoimmune mucocutaneous blistering disease associated with malignancy and induced by lymphoproliferative disorders [3].

## 2. Case

**Figure 1:**
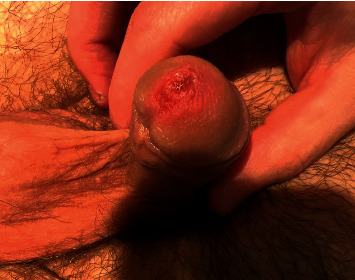
Lesion on the glans penis.

**Figure 2:**
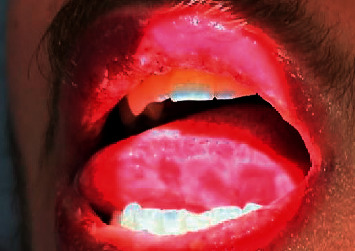
Ulcer on the tongue.

**Figure 3:**
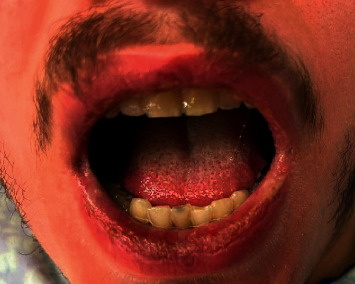
Mucocutaneous lesions involving the lower lip.

**Figure 4:**
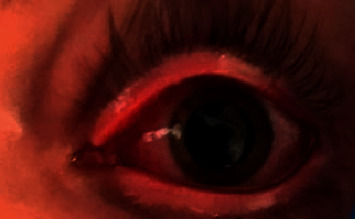
Left eye redness.

**Figure 5:**
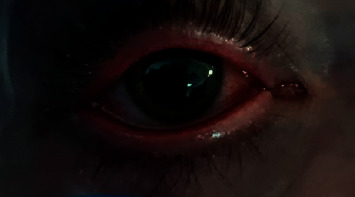
Right eye redness.

**Figure 6:**
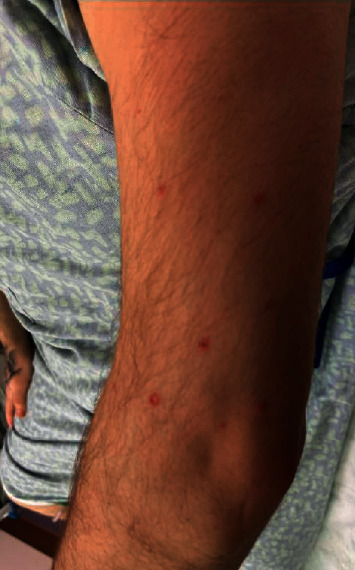
Maculopapular lesions on the left arm.

**Figure 7:**
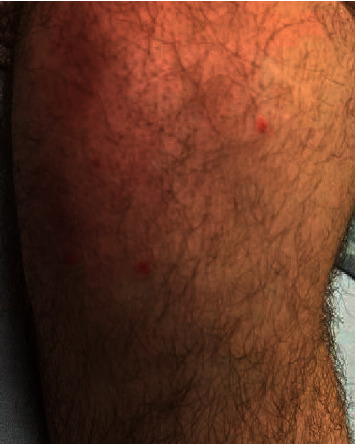
Maculopapular lesions on the right knee.

**Figure 8:**
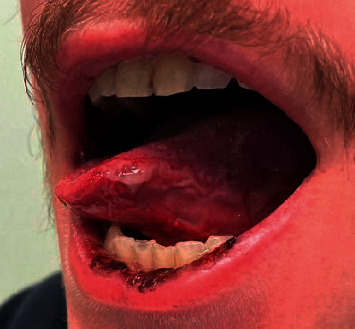
Resolving oral mucocutaneous lesions.

**Figure 9:**
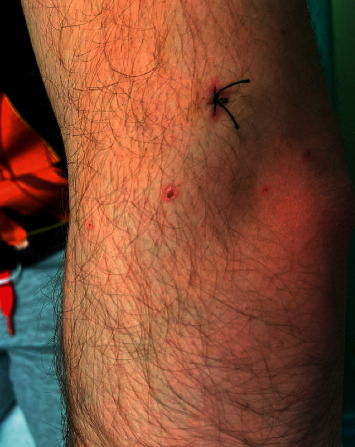
Resolving lesions on left arm involving lesion post-biopsy.

A 20-year-old healthy Caucasian male with no significant past medical history presented with painful mucocutaneous lesions involving the glans penis, oral sores, bilateral conjunctival redness, and painless rash on his arms and legs (Figures 1–7) over 3–5 days. The patient reported that his symptoms were preceded by upper respiratory infection-like symptoms with a runny nose, nonproductive cough, fatigue, sore throat, and a mild fever that resolved on its own 10 days prior. On exam, the patient had tachycardia with other vitals stable. The patient had difficulty urinating with the penile lesion at the urethral meatus causing urinary retention warranting the Foley insertion in the emergency department. He also received ceftriaxone and azithromycin for concerns of STDs. ENT performed nasopharyngeal scope and recommended steroids for laryngeal edema, no airway compromise was noted. He was admitted as an inpatient to the medicine service for further management. The Foley catheter was removed. The patient reported this was his third episode of developing oral lesions over the last 9 months. He had received steroids and antivirals from his PCP that resolved the oral lesions. He denied weight loss, joint pain, joint stiffness, photosensitivity, visual disturbances, personal history of blood clots, and family history of autoimmune diseases. The patient was a current smoker and in a monogamous relationship with his girlfriend who had been asymptomatic. Ophthalmology was consulted for bilateral conjunctival redness and reported no underlying ocular inflammation. Laboratory work came back with CBC showing leukocytosis of 15.9 with absolute neutrophilia, elevated inflammatory markers: ESR 52, CRP 115, ferritin 219. Rheumatology was consulted for concern for Behcet's disease. They performed a pathergy test that resulted negative over 24–48 hours. The patient also had a few episodes of diarrhea, hematuria, and proteinuria noted on UA during hospitalization. Abdominal ultrasound was obtained which did not show any acute abnormality or hepatosplenomegaly. Autoimmune workup including ANA specific by IFA, CCP, RF, and HLA B 51 resulted within normal limits. Infectious workup with CXR, mycoplasma, mono spot test, syphilis, HIV, COVID-19, HSV PCR, rapid strep, respiratory panel, chlamydia, gonorrhea, cold agglutinins, mycoplasma IgM, and IgG all resulted in negative. The patient was started on 0.6 mg colchicine twice daily and showed improvement. Skin biopsy was performed with the histologic differential diagnosis including a hypersensitivity reaction such as mycoplasma-induced rash and mucositis (MIRM), a drug or other hypersensitivity reaction, or viral exanthem while the direct immunofluorescent test showed linear fibrinogen deposition in the basement membrane zone. The patient was discharged home on the continuation of steroid taper and colchicine which was continued for 2 more weeks. On follow-up with rheumatology, primary care, ophthalmology, and urology patient was feeling significantly better with the rashes resolving (Figures 8 and 9), and inflammatory markers back to normal.

## 3. Discussion

*Mycoplasma pneumonia*e, a leading cause of community-acquired pneumonia, may cause extrapulmonary manifestations, including mucocutaneous eruptions, which have been reported in approximately 25 percent of pediatric patients and young adults [4]. MIRM should be suspected when a young patient presents with a mucosal or mucocutaneous eruption and a history of prodromal symptoms, including cough, malaise, and fever preceding the eruption by approximately one week [1]. MIRM is characterized by prominent mucositis, usually with sparse or even absent cutaneous involvement. Compared with SJS/TEN, MIRM has distinct pathophysiology, a milder course, and a generally good prognosis. Including MIRM in a broader category called “reactive infectious mucocutaneous eruption” (RIME) has been proposed. RIME describes mucocutaneous eruptions resulting from a variety of infectious triggers and differentiates infectious triggers, which are far more likely in children and adolescents, from drug triggers [5]. Proposed diagnostic criteria for classic cases of MIRM include mucocutaneous eruption with <10 percent body surface area involvement, involvement of two or more mucosal sites, presence of a few vesiculobullous lesions, or scattered, atypical, targetoid lesions, and clinical and laboratory evidence of *M. pneumoniae* infection [1]. Other authors have suggested adding young age to the diagnostic criteria, as MIRM is very rare in adults [6]. Confirmatory laboratory tests for *M. pneumoniae* include polymerase chain reaction (PCR) of pharyngeal swab and measurement of serum-specific immunoglobulin G (IgG), immunoglobulin M (IgM), and immunoglobulin A (IgA) titers [7]. Although PCR is highly sensitive and specific, it can remain positive for up to four months after infection, making it difficult to distinguish acute from past infection. IgM titers start to increase approximately seven to nine days after infection, peak at three to six weeks, and persist for months; IgG titers begin to rise and peak approximately two weeks after IgM titers and persist for years. Thus, as both IgM and IgG may be normal in the acute phase, documentation of titer increase in paired sera is needed for accurate serologic diagnosis. In our patients, all the diagnostic tests including cold agglutinins, PCR, IgM, and IgG came back negative. Patients with MIRM often have elevated acute phase reactants, including C-reactive protein and erythrocyte sedimentation rate [8]. Given the negative tests and systemic involvement, we were concerned about Behçet's disease since this was the third episode of oral ulcers over the last nine months. Behçet's disease is best diagnosed in the context of recurrent aphthous ulcerations along with characteristic systemic manifestations including ocular disease, especially hypopyon, pan uveitis, or retinal vasculitis; neurologic disease including characteristic central nervous system parenchymal findings; vascular disease, particularly pulmonary artery aneurysms, Budd-Chiari syndrome, and cerebral venous thrombosis; and patients with pathergy manifestations. Oral ulcerations also tend to be more frequent and severe in patients with Behçet's disease [9]. Although ophthalmology had ruled out ocular inflammation, concern for systemic disease persisted with the patient having episodes of diarrhea, hematuria, and proteinuria noted on UA raising concern for renal and GI involvement. There are no pathognomonic laboratory tests for Behçet's disease; as a result, the diagnosis is made based on the clinical findings. In the absence of other systemic diseases, the diagnosis of Behçet's disease is made based on patients having recurrent oral aphthae (at least three times in one year) plus two of the following clinical features. Recurrent genital aphthae (aphthous ulceration or scarring), eye lesions (including anterior or posterior uveitis, cells in vitreous on slit-lamp examination, or retinal vasculitis observed by an ophthalmologist), skin lesions (including erythema nodosum, pseudofolliculitis, papulopustular lesions, or acneiform nodules consistent with Behçet's disease), which indicate a positive pathergy test [10]. Pathergy is defined by a papule 2 mm or more in size developing 24 to 48 hours after oblique insertion of a 20-gauge needle 5 mm into the skin, generally performed on the forearm which was negative in our patient as per rheumatologist evaluation. Pathergy is less common in Northern European and North American patients. Thus, it has been suggested that other features might be substituted for pathergy in these populations, including aseptic meningoencephalitis, cerebral vasculitis, recurrent phlebitis, arthritis, synovitis, epididymitis, or focal bowel ulceration [11]. HLA-B*∗*51 remains the most important genetic factor in Behçet's disease, despite the recent identification of several susceptibility genes [12]. Some patients do not meet these criteria in whom the diagnosis of Behçet's disease is still made and establishing the diagnosis in such patients is much more difficult. Thus, our patient with a suspected diagnosis of Behçet's disease was started on colchicine. A progressive and painful mucositis is uniformly present in patients with PNP that is erosive in nature. Oral involvement is the most common and initial manifestation in most patients who develop painful, erosive stomatitis that characteristically includes the involvement of the tongue [13]. Investigations to diagnose PNP include checking for systemic complications (to identify tumors) and skin biopsies [3]. With the diagnosis still in question, a skin biopsy was performed which resulted in MIRM. A mucosal or cutaneous biopsy is not routinely performed for the diagnosis of MIRM. However, a skin biopsy including direct immunofluorescence should be performed if an autoimmune blistering disorder is being considered in the differential diagnosis. The patient had already received treatment for MIRM empirically in the ED with antibiotics. Most mycoplasma infections are self-limiting; however, treatment with antibiotics is useful if the extrapulmonary manifestation is due to a direct invasion of the organism. Macrolide, tetracycline, or fluoroquinolone classes of antibiotics are preferred, considering the age of the patient and local antibiotic resistance patterns. The duration of antibiotics in pulmonary infection is usually 5 to 7 days, but in extrapulmonary infection undetermined. The use of steroids is controversial, but studies have shown benefits in the setting of immune-mediated manifestations [14]. Colchicine and steroid taper were continued for up to two weeks, which helped as an anti-inflammatory. The rheumatologist stopped colchicine at the clinic visit with HLA-B*∗*51 also resulted negative. Health care providers should be educated to recognize MIRM and differentiate it from autoimmune diseases like Behçet's disease. Making a correct diagnosis is imperative to reassure patients and to avoid further costly referrals and additional unnecessary treatment.
